# A molecular signature for the G6PC3/SLC37A2/SLC37A4 interactors in glioblastoma disease progression and in the acquisition of a brain cancer stem cell phenotype

**DOI:** 10.3389/fendo.2023.1265698

**Published:** 2023-11-16

**Authors:** Sima Torabidastgerdooei, Marie-Eve Roy, Borhane Annabi

**Affiliations:** Laboratoire d’Oncologie Moléculaire, Centre de recherche CERMO-FC, Département de Chimie, Université du Québec à Montréal, Montreal, QC, Canada

**Keywords:** glioblastoma, glucose-6-phosphatase system, G6PC3, G6PT, SLC37A2, SLC37A4, cancer stem cells, metabolic reprogramming

## Abstract

**Background:**

Glycogen plays an important role in glucose homeostasis and contributes to key functions related to brain cancer cell survival in glioblastoma multiforme (GBM) disease progression. Such adaptive molecular mechanism is dependent on the glycogenolytic pathway and intracellular glucose-6-phosphate (G6P) sensing by brain cancer cells residing within those highly hypoxic tumors. The involvement of components of the glucose-6-phosphatase (G6Pase) system remains however elusive.

**Objective:**

We questioned the gene expression levels of components of the G6Pase system in GBM tissues and their functional impact in the control of the invasive and brain cancer stem cells (CSC) phenotypes.

**Methods:**

*In silico* analysis of transcript levels in GBM tumor tissues was done by GEPIA. Total RNA was extracted and gene expression of *G6PC1-3* as well as of *SLC37A1-4* members analyzed by qPCR in four human brain cancer cell lines and from clinically annotated brain tumor cDNA arrays. Transient siRNA-mediated gene silencing was used to assess the impact of TGF-β-induced epithelial-to-mesenchymal transition (EMT) and cell chemotaxis. Three-dimensional (3D) neurosphere cultures were generated to recapitulate the brain CSC phenotype.

**Results:**

Higher expression in *G6PC3*, *SLC37A2*, and *SLC37A4* was found in GBM tumor tissues in comparison to low-grade glioma and healthy tissue. The expression of these genes was also found elevated in established human U87, U251, U118, and U138 GBM cell models compared to human HepG2 hepatoma cells. *SLC37A4/G6PC3*, but not *SLC37A2*, levels were induced in 3D CD133/SOX2-positive U87 neurospheres when compared to 2D monolayers. Silencing of *SLC37A4*/*G6PC3* altered TGF-β-induced EMT biomarker SNAIL and cell chemotaxis.

**Conclusion:**

Two members of the G6Pase system, G6PC3 and SLC37A4, associate with GBM disease progression and regulate the metabolic reprogramming of an invasive and CSC phenotype. Such molecular signature may support their role in cancer cell survival and chemoresistance and become future therapeutic targets.

## Graphical Abstract

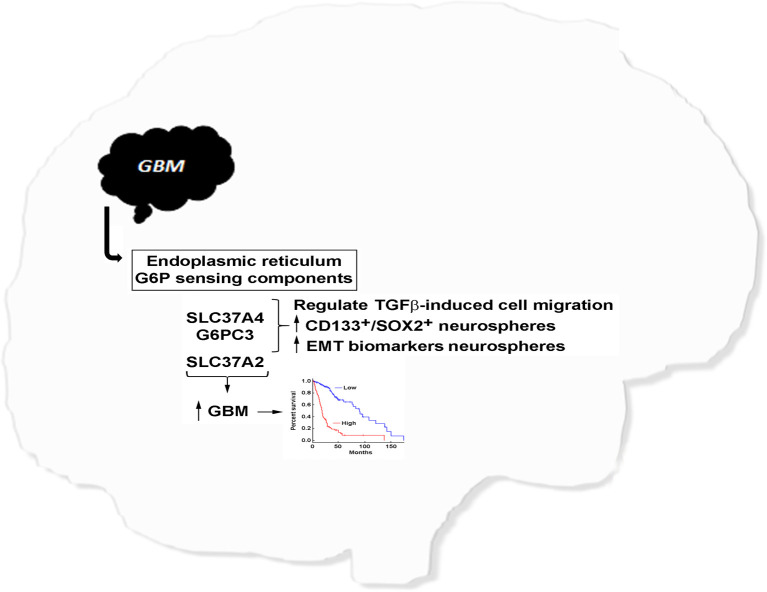

## Introduction

The metabolic reprogramming of cancer cells is a key contributing factor to tumorigenesis enabling rapid proliferation, survival, invasion, metastasis, resistance to treatments, and other processes associated with carcinogenesis (1–3). Reprogramming energy metabolism is also an important adaptive mechanism exploited by hypoxic solid tumors to increase cell survival within a low oxygen tumor microenvironment (4–6). As such, hypoxic cancer cells exhibit the Warburg effect allowing them to use glycolysis to convert glucose into pyruvate as their primary source of energy production, even in the presence of oxygen (aerobic glycolysis) (7, 8). In cases of nutrient deficiency, such as lack of glucose, this method of energy production may not be efficient. Thus, as cancer cells constantly activate additional metabolic reprogramming to complete their energy needs, a better understanding of glucose metabolism and enzymatic systems involved will help design better cancer therapeutic strategies (9).

Of particular interest, the flux in glucose/glucose-6-phosphate (G6P) can be sensed, in part, through components system of the glycogen metabolism which appear dysregulated in a wide variety of malignancies (10). Indeed, the levels of glycogen were demonstrated to be particularly high in breast, kidney, uterus, bladder, ovary, skin, and brain cancer cell lines (11). The glucose-6-phosphatase (G6Pase) system, located at the endoplasmic reticulum (ER) membrane, has been identified as a significant enzymatic system in the regulation of glucose homeostasis and in glycogenolysis (12, 13), and deficiencies responsible for type I glycogen storage disease (14). It is composed of two main components, the G6P translocase (G6PT/SLC37A4) and the G6Pase catalytic subunit (G6PC). SLC37A4 senses and transports G6P from the cytoplasm to the ER lumen where it is hydrolyzed by the G6PC (15, 16), the latter activity being linked to glycogen turnover in cancer cells (17). In hepatocellular carcinoma (HCC), G6PC expression (18–20) and activity (18) were downregulated compared with adjacent tumor-free tissues, and similar results were obtained in renal cell carcinoma (RCC) (20, 21). In contrast, G6PC expression was enhanced in glioblastoma (GBM), a highly malignant brain cancer, compared to the noncancerous human cortex (22). Tumor-initiating cells isolated from GBM showed an upregulation of G6PC by the glycolysis inhibitor 2-desoxyglucose (2DG) (22). G6PC silencing reduced proliferation and migration of GBM cells and invasion *in vivo*, which was especially pronounced after 2DG treatment and recovery. Mechanistically, G6PC silencing is believed to lead to glycogen accumulation and to result in reduced activation of AKT (protein kinase B) (22).

High G6PC expression was found to be associated with poor overall and disease-free survival in ovarian cancer (23). Here again, silencing G6PC in ovarian cancer was associated with an accumulation of glycogen, a downregulation of PYGL (a regulator of glycogenolysis), a reduction in cell growth, and in an inhibition of the epithelial-to-mesenchymal transition (EMT) (23). Interestingly, glycogen accumulation is also a key initiating oncogenic event during liver malignant transformation where G6PC is frequently downregulated to augment glucose storage in pre-malignant cells (24). Consistently, the elimination of glycogen accumulation abrogated liver growth and cancer incidence, whereas increasing glycogen storage appeared to accelerate tumorigenesis. It is further hypothesized that cancer-initiating cells adopt a glycogen storing mode to augment tumor incidence.

The G6PC family consists of three members: G6PC1 (G6PCα), G6PC2, and G6PC3 (G6PCβ) (25). Despite their distinct expression patterns and associated diseases, all three members can hydrolyze G6P (26), although the hydrolyzing activity of G6PC2 and G6PC3 is believed to be much lower than that of G6PC1 (25). In addition, the SLC37A family members are ER-associated sugar-phosphate/phosphate (P(i)) exchangers consisting of four members: SLC37A1, SLC37A2, SLC37A3, and SLC37A4. Aside from SLC37A3 which function is unknown, the other three members play roles in the final step of the gluconeogenic and glycogenolytic pathways (16). However, unlike SLC37A4, which is functionally coupled with G6PCα and G6PCβ to hydrolyze G6P, SLC37A1 and SLC37A2 cannot couple with these two proteins to function (27).

Several underestimated roles for the SLC37A4 as a potential regulator of human U87 GBM cancer cells’ invasive phenotype and of angiogenic processes were documented in brain endothelial cells (28–30). In addition, increased *SLC37A4* transcriptional regulation under hypoxic culture conditions was also reported to require hypoxia inducible factor (HIF)-1α (31). SLC37A4’s potential role included regulation of calcium-mediated signaling, which is known to control cancer cell proliferation, cell cycle division, extracellular matrix degradation, and response to growth factors (32, 33).

While GBM is a highly aggressive form of brain cancer (34), recent advances in our understanding of their metabolism suggests that it is highly heterogeneous, and that cancer stem cells (CSC), a small subset of all cancer cells, may further exhibit specific metabolic traits that could play a significant role in anticancer therapy failure (35). The objectives of the current study were to address, as a first investigatory step at the transcriptional level, to what extent the expression of *G6PC* and *SLC37A* members were regulated in *i)* the transition from healthy brain to low-grade glioma, then to GBM tissues, and *ii)* in established human GBM cell line models. Moreover, their contribution in *iii)* the chemotactic response and the acquisition of a CSC phenotype were also explored. Understanding CSC biology and metabolic reprogramming involving the G6Pase components could become keys to optimizing anticancer treatments (36).

## Materials and methods

### Materials

Sodium dodecyl sulfate (SDS) and bovine serum albumin (BSA) were purchased from Sigma-Aldrich Canada (Oakville, ON). Cell culture media was obtained from Life Technologies (Burlington, ON). Electrophoresis reagents were purchased from Bio-Rad (Mississauga, ON). All other reagents were from Sigma-Aldrich Canada.

### *In silico* analysis of transcripts levels in clinical glioblastoma and low-grade glioma tissues

A Gene Expression Profiling Interactive Analysis (GEPIA) web server was used to analyze the RNA sequencing expression data of glioblastoma tumors (GBM, n = 163) vs. healthy tissue (n = 207), and of low-grade glioma (LGG, n = 251) vs. healthy tissue (n = 207) from the TCGA and the normal brain tissue in Genotype-Tissue Expression (GTEx) databases (37). GEPIA provides customizable functions such as tumor/normal differential expression analysis, profiling according to cancer types or pathological stages, patient survival analysis, similar gene detection, correlation analysis, and dimensionality reduction analysis (http://gepia.cancer-pku.cn/detail.php, accessed on July 5^th^, 2023). One-way ANOVA was used for differential analysis of gene expression, using disease states (GBM, LGG, or normal) as variables for the box plots.

### Prognostic value of *G6PC3, SLC37A2*, and *SLC37A4* in glioblastoma patients

The prognostic value of mRNA level of *G6PC3*, *SLC37A2*, and *SLC37A4* factors in GBM patients was analyzed by GEPIA (37). For each of the three genes tested, browsing of human gene-expression fingerprints was retrieved from a web-based database containing a large number of high-quality data sets in GBM tissues. Log-rank tests for overall survival analyses were used.

### Protein-protein interaction

The associative relationships of G6PC3 and of SLC37A2 were retrieved from the STRING v11 database (https://www.string-db.org/) to identify and build protein-protein interaction networks (38), with a confidence score setting of 0.4, and the maximum number of interactions to show was no more than 10.

### Cell culture

The human U87 (HTB-14), U118, U138, and U251 glioblastoma cell lines, as well as the human HepG2 hepatoma cell line, were from American Type Culture Collection (Manassas, VA). They were all maintained in Eagle’s Minimum Essential Medium (Wisent, 320-006CL) containing 10% (v/v) calf serum (HyClone Laboratories, SH30541.03), 2 mM glutamine, 1 mM sodium pyruvate (Sigma-Aldrich Canada, P2256), 100 units/ml penicillin and 100 mg/ml streptomycin (Wisent, 250-202-EL). Cells were incubated at 37°C with 95% air and 5% CO_2_. Neurosphere formation was performed as follows: 80-90% adherent U87 monolayer cells were trypsinized and plated in low adhesion 24-well plates (Corning Costar, Corning, NY, USA) at a density of 2x10^5^ cells/mL in complete media for 24-72 hours. Then, the supernatant was removed, and serum-free EMEM supplemented with 10 ng/mL human basic fibroblast growth factor (Gibco, Thermo Fisher, 13256029), 20 ng/mL human epidermal growth factor (Gibco, Thermo Fisher, PHG0315), 5 μg/mL insulin (Sigma Aldrich Corp, I3536), and BSA (Sigma Aldrich Corp, A9418-5G), at 4% was carefully added to the dishes. Spheroids were defined as rounded aggregates of cells with a smooth surface and poor cell-to-cell definition. Perimeters of 30-70 spheroids/flask were assessed for each experimental condition performed in triplicate and derived from three independent experiments.

### TissueScan cDNA arrays of grades I-IV brain tumor tissues

TissueScan™ cancer and normal tissue cDNA arrays were purchased from OriGene (Rockville, MD), covering 43 clinical samples of the four stages of brain cancer as well as normal tissues, and were used to assess *G6PC3*, *SLC37A2*, and *SLC37A4* gene expression levels according to the manufacturer’s recommendations. Tissue cDNAs in each array were synthesized from high-quality total RNAs of pathologist-verified tissues, normalized and validated with ß-actin in two sequential qPCR analyses, and accompanied by clinical information for 18 WHO grade I, 11 WHO grade II, 10 WHO grade III, and 2 WHO grade IV brain tumors.

### Total RNA isolation, cDNA synthesis and real-time quantitative RT-PCR

Total RNA was extracted from cell monolayers using TriZol reagent (Life Technologies, 15596-018). For cDNA synthesis, 2 μg of total RNA was reverse transcribed using a high-capacity cDNA reverse transcription kit (Applied Biosystems, 4368814). cDNA was stored at -80°C prior to PCR. Gene expression was quantified by real-time quantitative PCR using Sso Fast EvaGreen Supermix (Bio-Rad). DNA amplification was carried out using a CFX Connect Real-Time System (Bio-Rad) and product detection was performed by measuring the binding of the fluorescent dye EvaGreen to double-stranded DNA. The following QuantiTect primer sets were provided by QIAGEN: G6PC1 (Hs_G6PC_1_SG QT00031913), G6PC2 (Hs_G6PC2_va.1_SG QT01664152), G6PC3 (Hs_G6PC3_1_SG QT00033453), SLC37A1 (SLC37A1_1_SG QT00073094), SLC37A2 (Hs_SLC37A2_1_SG QT00056203), SLC37A3 (Hs_SLC37A3_1_SG QT00057148), SLC37A4 (Hs_SLC37A4_1_SG QT00024325), GAPDH (Hs_GAPDH_2_SG QT01192646), β-actin (Hs_Actb_2_SG QT01680476) and PPIA (Hs_PPIA_4_SG QT01866137). The relative quantities of target gene mRNA compared against two internal controls chosen from GAPDH, β-actin or PPIA RNA, were measured by following a ΔCT method employing an amplification plot (fluorescence signal *vs*. cycle number). The difference (ΔCT) between the mean values in the triplicate samples of the target gene and those of GAPDH and β-actin mRNAs were calculated by CFX manager Software version 2.1 (Bio-Rad) and the relative quantified value (RQV) was expressed as 2^-ΔCT^.

### Transfection method and RNA interference

For gene silencing experiments, U87 glioblastoma cells were transiently transfected with siRNA sequences using Lipofectamine-2000 transfection reagent (Thermo Fisher Scientific, Waltham, MA, USA). Gene silencing was performed using 20 nM siRNA against G6PC3 (Hs_G6PC3_5 siRNA, SI02659363), SLC37A2 (Hs_SLC37A2_7 siRNA, SI04151840), SLC37A4 (Hs_SLC37A4_3 siRNA, SI00724213), or scrambled sequences (AllStar Negative Control siRNA, 1027281). The above small interfering RNA and mismatch siRNA were all synthesized by QIAGEN and annealed to form duplexes. Gene silencing efficacy was assessed by RT-qPCR as described above.

### Real-time cell migration assay

Experiments were carried out using the Real-Time Cell Analyser (RTCA) Dual-Plate (DP) Instrument and the xCELLigence system (Roche Diagnostics, QC), following the instructions of the supplier. U87 cells were transfected with 2 nM siRNAs (Control and G6PC3) as described above. After transfection, 25,000 cells per well were seeded in a CIM-plate 16 (Roche Diagnostics) and incubated at 37°C under a humidified atmosphere containing 5% CO_2_ for 24 hours. Prior to cell seeding, the underside of each well in the upper chamber was coated with 0.15% gelatin in PBS and incubated for 1 hour at 37°C. The lower chamber was filled with serum-free medium. The upper chamber of each well was filled with 250,000 cells. After 30 min of adhesion, cell migration was monitored every 5 min for 20 hours. The impedance value was measured by the RTCA DP Instrument and expressed as an arbitrary unit called the Cell Index. Each experiment was performed in quadruplicate wells.

### Statistical data analysis

Data are representative of three or more independent experiments. Statistical significance was assessed using Student’s unpaired t-test or 1-way ANOVA with a Dunnett post-test. Probability values of less than 0.05 were considered significant, and an asterisk (*) identifies such significance in the figures.

## Results

### Increased gene expression *of G6PC3, SLC37A2*, and *SLC37A4* in clinically-annotated glioblastoma tumor tissues

**Figure 1:**
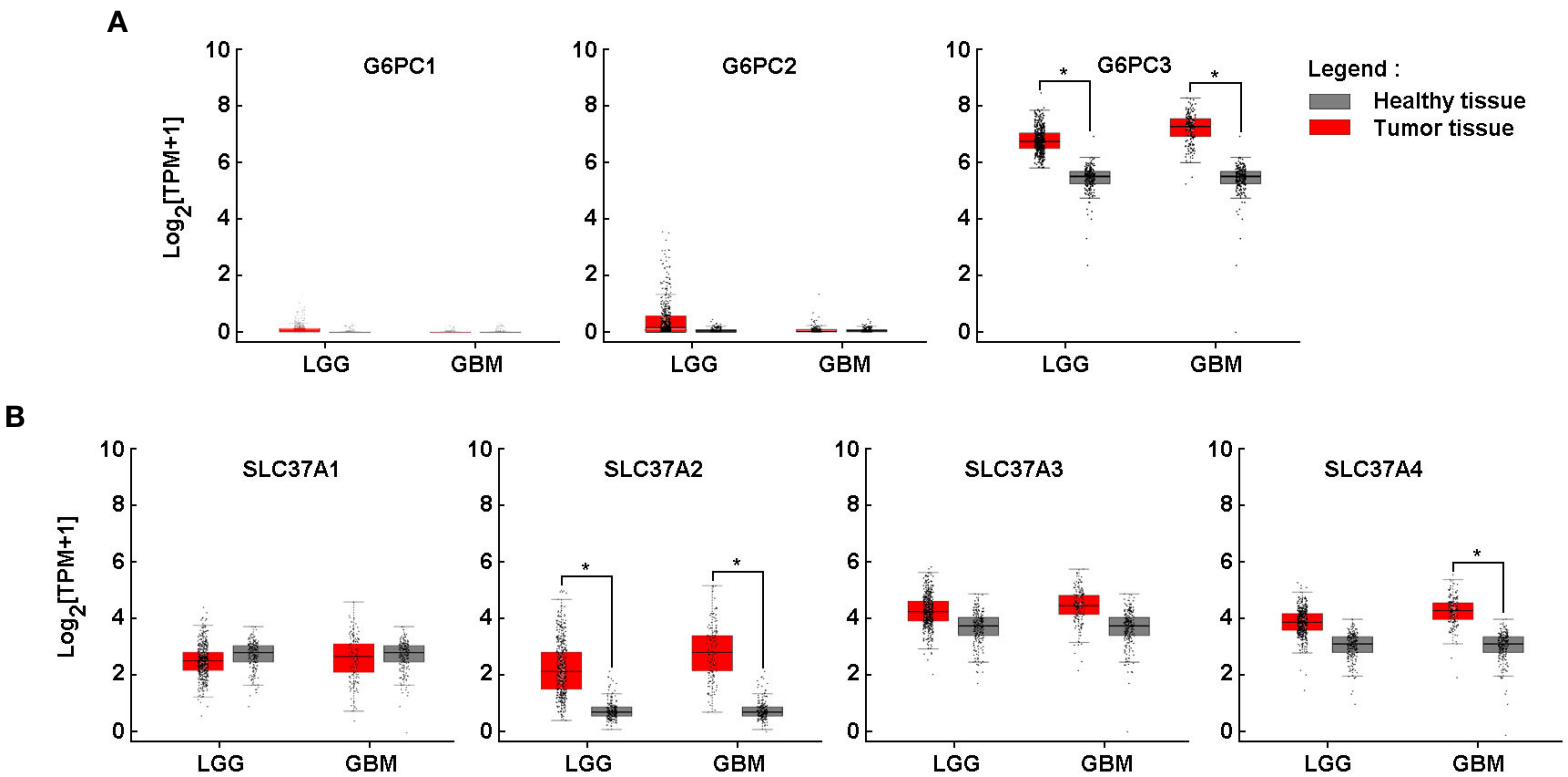
Increased gene expression of *G6PC3*, *SLC37A2*, and *SLC37A4* in clinically-annotated glioblastoma tumor tissues. In silico analysis of transcript levels was performed for **(A)** three members of the G6PC family (G6PC1, G6PC2, G6PC3) and **(B)** four members of the SLC37A family (SLC37A1, SLC37A2, SLC37A3, SLC37A4) using RNA extracted from clinical samples from glioblastoma (GBM) and low-grade glioma (LGG) (red boxes) and compared to healthy tissue (grey boxes). (*p <0.05).

As previously mentioned in the Methods section, an *in silico* differential analysis of *G6PC* (*G6PC1*, *G6PC2*, *G6PC3*) (**Figure 1A**) and *SLC37A* (*SLC37A1*, *SLC37A2*, *SLC37A3*, *SLC37A4*) (**Figure 1B**) family members transcript levels was conducted on clinical samples from glioblastoma (GBM) and low-grade glioma (LGG), and compared to healthy brain tissues. The expression of *G6PC1* and *G6PC2* were very low in both LGG and GBM samples (**Figure 1A**, left and middle panels), whereas that of *G6PC3* was high in both tissues and increased significantly when compared to healthy brain tissue (**Figure 1A**, right panel). All four *SLC37A* members were significantly expressed in LGG and GBM tissues (**Figure 1B**, red boxes). When compared to healthy tissues, only *SLC37A2* was significantly increased in LGG and GBM samples, whereas the expression of *SLC37A4* increased only in GBM samples and not in LGG (**Figure 1B**). This suggests that these three gene candidates may possibly serve as biomarkers of brain cancer disease progression. Their transcript expression was next further assessed in tumor tissues cDNA arrays using clinically-annotated GBM samples from all four stages.

### High G6PC3, SLC37A2, and SLC37A4 gene expression correlates with poor prognosis in GBM patients

**Figure 2:**
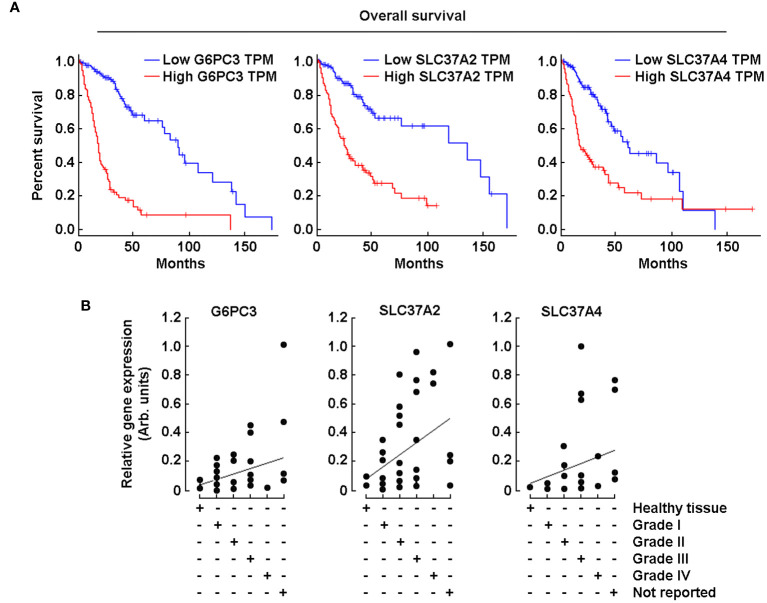
High *G6PC3*, *SLC37A2*, and *SCL37A4* gene expression correlates with poor prognosis in GBM patients. **(A)** Kaplan-Meier analysis was performed using transcriptional programs addressing a database of gene expression profiles from healthy and malignant brain cancer as described in the Methods section. The three panels show a survival plot based on a high-quality data set displaying a full analysis of *G6PC3*, *SLC37A2*, and *SCL37A4*. Blue lines show patients with gene expression below median levels (low expression), whereas red lines show patients with gene expression above median (high expression). **(B)** TissueScan™ brain cancer and normal tissue cDNA arrays from 43 clinical samples covering four stages of brain cancer were used to assess *G6PC3*, *SLC37A2*, and *SCL37A4* gene expression levels.

With the same online analytical tool, a survival analysis was performed of the three genes that showed increased expression of their transcript levels in tumor tissues (**Figure 1**, red box). When the analysis was performed with high expression of these three genes, the overall survival rate was reduced significantly (**Figure 2A**, red lines). Further, RT-qPCR analysis was performed using brain tissue scan arrays as described in the Methods section. The results show that compared to healthy brain tissue, as the grades of GBM increased, the expression of these three genes was also induced (**Figure 2B**).

### Protein-protein interaction network predicts G6PC3 interrelationship with SLC37A4, but not SLC37A2

**Figure 3:**
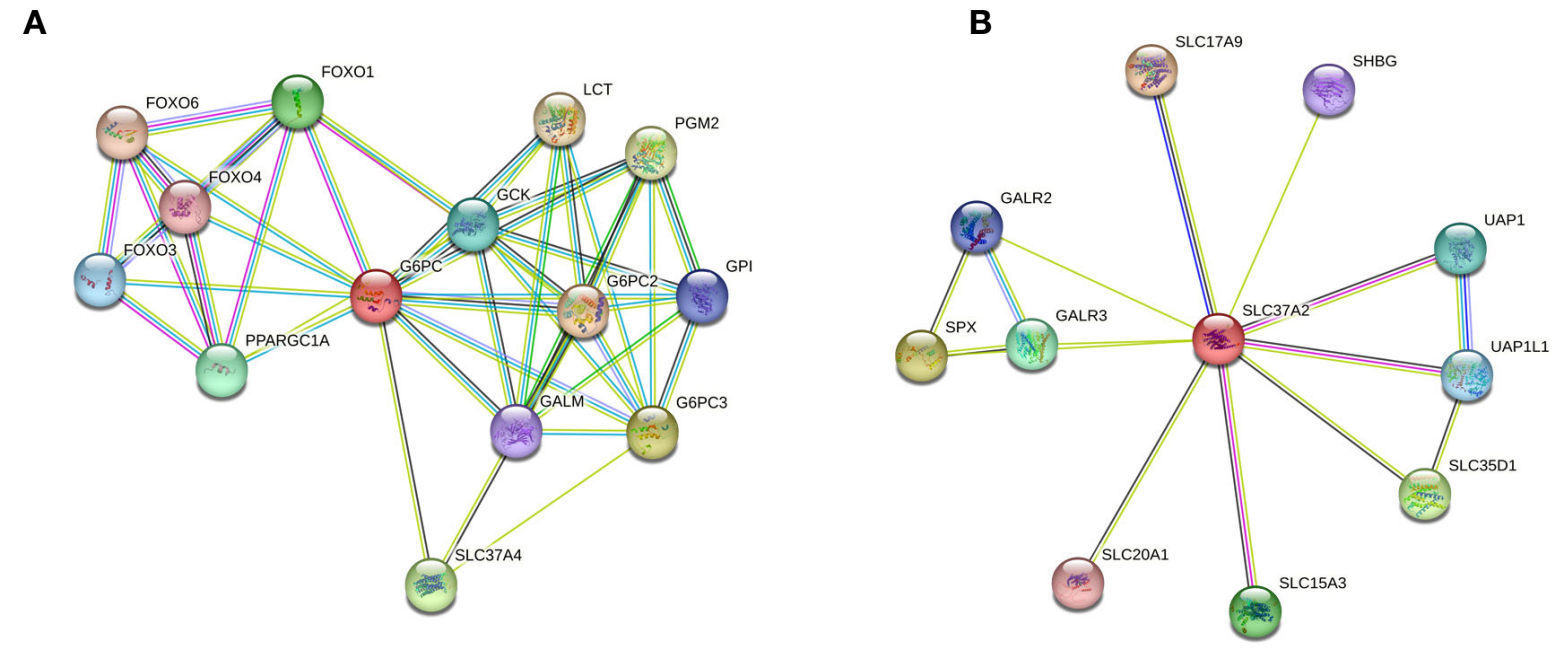
Protein-protein interaction (PPI) network predicts G6PC3 interrelationship with SLC37A4, but not SLC37A2. Indirect target proteins of the core **(A)** G6PC3- and **(B)** SLC37A2-associated network were built by STRING database as described in the Methods section.

A protein-protein interaction (PPI) network of G6PC3 was constructed by using the STRING database (https://string-db.org/) and was used to predict and analyze potential or existing PPI. Indirect target proteins of G6PC3 were retrieved from STRING as described in the Methods section and predicted G6PC3 interrelationship with potential biomarkers involved in the G6Pase system, namely G6PC1 and SLC37A4 (**Figure 3A**). Given that no relationship was found between G6PC3 and SLC37A2, an independent interaction network analysis was performed which confirmed that predicted PPI network did not involve components of the G6Pase system (**Figure 3B**).

### Expression of *G6PC3*, *SLC37A2*, and *SLC37A4* is increased in several human glioblastoma cell lines

**Figure 4:**
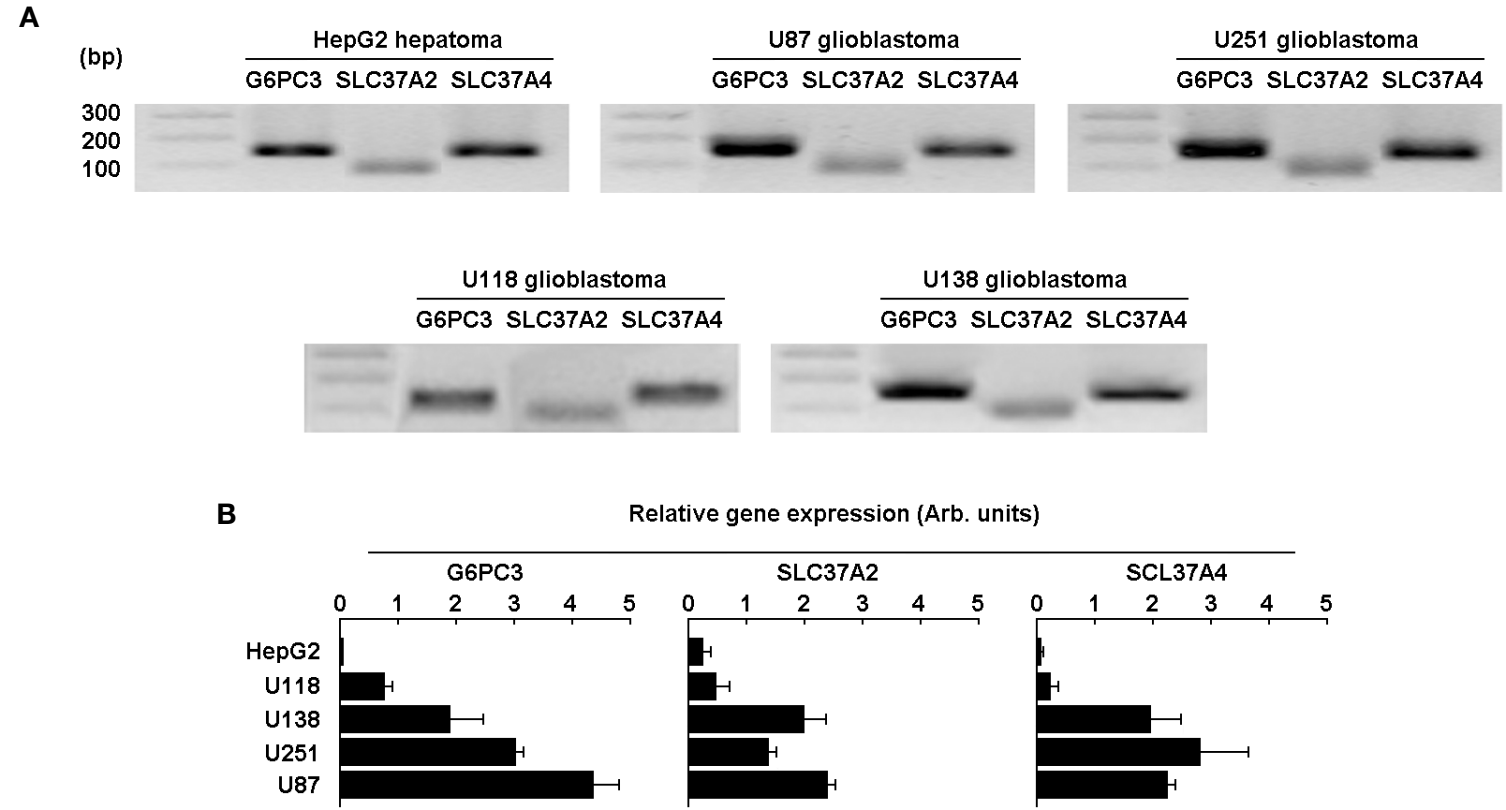
Expression of the G6PC3, SLC37A2, and SLC37A4 are increased in several human glioblastoma cell lines.> Total RNA was extracted from four different human glioblastoma cell lines (U87, U118, U251, and U138) and the gene expression level for *G6PC3*, *SLC37A2*, and *SLC37A4* analyzed by RT-qPCR and compared to the human HepG2 hepatoma cell line. **(A)** Primers validation and single amplicon products were confirmed using agarose gel electrophoresis for all cell lines tested. **(B)** The gene expression levels of the three selected genes was analyzed and quantified by qPCR as described in the Methods section. Triplicates from a representative quantification, out of three independent experiments, is shown.

To further validate the *in silico* analysis and cDNA array results, total RNA was extracted from four different human glioblastoma cell lines (U87, U251, U118, U138) and from one human hepatoma cell line (HepG2). Primers and RT-qPCR results were validated with agarose gel electrophoresis, which showed a single amplicon product for each of the genes amplified (**Figure 4A**). Next, qPCR analysis revealed that the expression of *G6PC3*, *SLC37A2*, and *SLC37A4* was considerably higher in all four glioma cell lines tested when compared to HepG2 cells (**Figure 4B**).

### *G6PC3* and *SLC37A4* levels are induced in CD133/SOX2-positive U87-derived neurospheres

**Figure 5:**
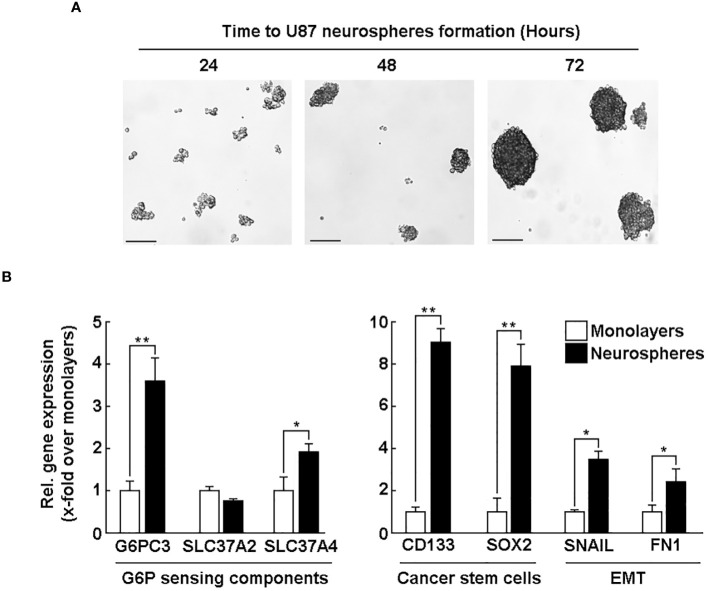
*G6PC3* and *SLC37A4* levels are induced in CD133/SOX2-positive U87-derived neurospheres. Human U87 glioblastoma cell monolayers were cultured with the Tumorsphere Medium Xf with SupplementMix for up to 72 hours. **(A)** Phase contrast pictures were taken to monitor spheroids formation at the indicated time. 4x magnification; scale bar = 90 μm. **(B)** Total RNA was extracted from either adherent monolayers (white bars) or from neurospheres (black bars) cultured for 72 hours. RT-qPCR analysis was next used to study the expression of *G6PC3, SLC37A2, SLC37A4*, CSC markers *CD133* and *SOX2*, as well as EMT markers *FN1* and *Snail*. Triplicates from a representative quantification, out of three independent experiments, is shown. (*p <0.05, **p <0.01).

Neurospheres culture conditions are known to recapitulate, in part, the cancer stem cells (CSC) phenotype (36, 39). U87 glioblastoma 3D neurosphere cultures appeared to reach maturation at 72 hours (**Figure 5A**) as described previously (40). To address how such phenotype impacted the *G6PC3*, *SLC37A2*, and *SLC37A4* transcript levels, total RNA was isolated, and qPCR analysis was performed in comparison to 2D cell monolayers. The results revealed increased expression in *G6PC3* and *SLC37A4* transcripts in neurospheres as compared to adherent cells, while *SLC37A2* levels remained unaltered (**Figure 5B**, left panel). In addition, the expression level of the CSC markers *CD133*, *SOX2*, and of epithelial-to-mesenchymal transition (EMT) markers *FN1* and *SNAIL* were also increased in neurospheres compared to adherent cells (**Figure 5B**, right panel). CD133, which is neurospheres positive control, is associated with resistance to *in vitro* chemotherapy and therefore may relate G6PC3 (G6PCβ) and SLC37A4 (G6PT) to some chemoresistance and invasive molecular signature of GBM-derived CSC.

### Silencing of G6PC3 and SLC37A4 alters the acquisition of a CSC phenotype and the expression of EMT-related biomarkers

**Figure 6:**
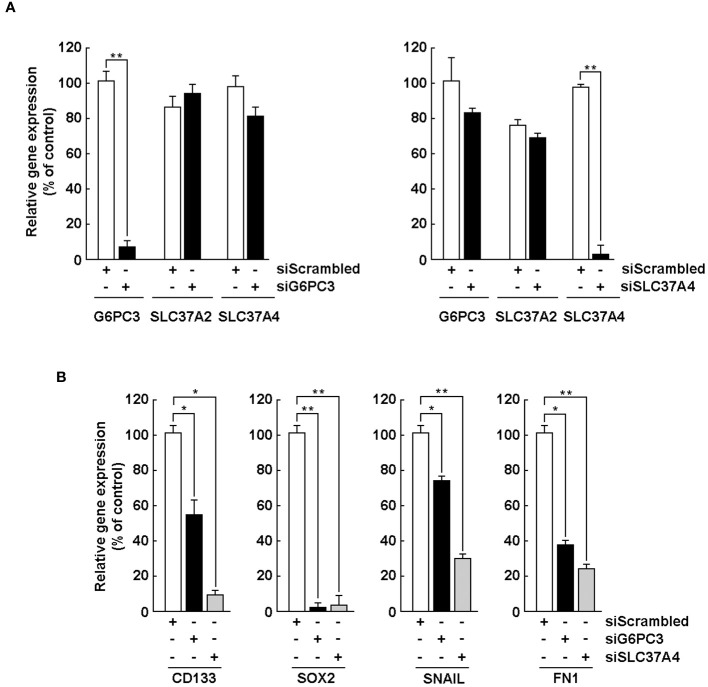
Silencing of G6PC3, SLC37A2, and SLC37A4 alters the acquisition of a CSC phenotype and expression of EMT markers. **(A)** U87 glioblastoma monolayers were transiently transfected with a scrambled sequence (siScrambled, white bars) or siRNA directed against G6PC3 (siG6PC3, left panel, black bars), or SLC37A4 (siSLC37A4, right panel, black bars). Total RNA was then extracted, and gene silencing efficiency validated using RT-qPCR. **(B)** After 24 hours of transfection, U87 cells were cultured with the Tumorsphere Medium Xf with SupplementMix for 72 hours. Total RNA was extracted from neurospheres and selected CSC and EMT markers analyzed by RT-qPCR in siScrambled (white bars), siG6PC3 (black bars), and siSLC37A4 (grey bars). (*p <0.05, **p <0.01, n = 3 independent experiments).

We next wished to address the potential crosstalk between the acquisition of CSC phenotype and the corresponding increase in G6PC3 and SLC37A4 expression upon neurospheres formation. U87 glioblastoma monolayer cells were transiently transfected with a scrambled sequence (siScrambled, **Figure 6A**, white bars) or siRNA directed against *G6PC3* (siG6PC3, **Figure 6A**, left panel, black bars), or *SLC37A4* (siSLC37A4, **Figure 6A**, right panel, black bars). Total RNA was then extracted, and the specificity of gene silencing confirmed for each repressed gene using RT-qPCR. Next, transfected U87 cells were cultured with the Tumorsphere Medium Xf with SupplementMix for 72 hours to generate neurospheres. Total RNA was again extracted from neurospheres and selected CSC (CD133, SOX2) and EMT (FN1, SNAIL) markers analyzed by RT-qPCR in siScrambled (**Figure 6B**, white bars), siG6PC3 (**Figure 6B**, black bars), and siSLC37A4 (**Figure 6B**, grey bars). In all the conditions tested, the expression of *CD133*, *SOX2*, *SNAIL*, and *FN1* was all prevented in neurospheres upon either *G6PC3* or *SLC37A4* repression. This could suggest that metabolic reprogramming involving these two genes are important factors that contribute to the acquisition of a CSC phenotype and induction of EMT biomarkers during neurospheres formation possibly through their respective G6P sensing (SLC37A4) and/or hydrolysing (G6PC3) activities.

### Evidence for G6PC3 and SLC37A4 involvement in the chemotactic response of U87 glioblastoma cells to TGF-β

**Figure 7:**
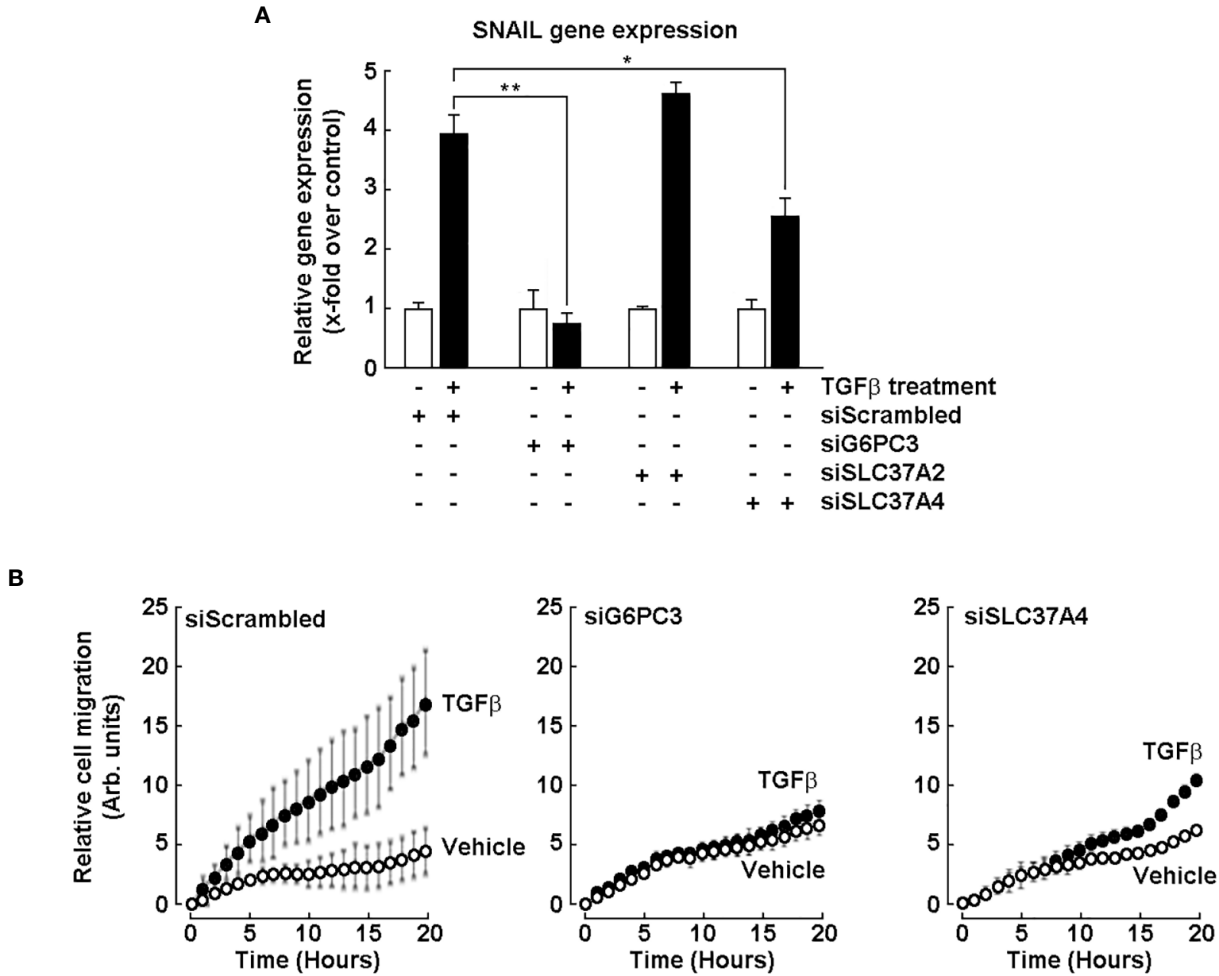
Evidence for G6PC3 and SLC37A4 involvement in the chemotactic response of U87 glioblastoma cells to TGF-β. Transient siRNA-mediated *G6PC3*, *SLC37A2*, and *SLC37A4* gene silencing was performed in U87 glioblastoma cells. **(A)** Cells’ response to 10 nM TGF-β treatment for 24 hours was next monitored by RT-qPCR through the assessment of SNAIL gene expression. (*p <0.05, **p <0.01, n = 3 independent experiments). **(B)** Cell chemotaxis was assessed in unstimulated (vehicle, open circles) or in response to 10 nM TGF-β (closed circles) as described in the Methods section.

Induced expression of EMT biomarkers SNAIL and FN1 documented above appears to play a role in the acquisition of a CSC phenotype in neurospheres. We thus questioned whether any early signaling events could be mimicked in such cellular response to transforming growth factor-beta (TGF-β), a known potent EMT inducer in glioblastoma cells (41, 42), and how G6PC3 and SCL37A4 would be involved. U87 monolayer cells were transiently transfected with specific siRNA to repress the expression of *G6PC3*, *SLC37A2*, and *SLC37A4*, or with a scrambled siRNA (Control). Cells were then challenged with 10 nM TGF-β for 24 hours. Total RNA was extracted, followed by RT-qPCR assessment of *SNAIL* gene expression. TGF-β effectively induced *SNAIL* in siScrambled-transfected cells, and this was significantly inhibited upon *G6PC3* and *SLC37A4* gene silencing, but not in cells where *SLC37A2* was silenced (**Figure 7A**). This suggested that G6PC3 would potentially also regulate additional TGF-β-mediated cellular events in U87 cells. Thus, TGF-β-induced cell chemotaxis was next assessed. Interestingly, whereas TGF-β effectively triggered migration in siScrambled-transfected cells (**Figure 7B**, left panel), whereas cells were found unresponsive to TGF-β when *G6PC3* or *SLC37A4* were silenced (**Figure 7B**, middle and right panels).

## Discussion

Glioblastoma (GBM) is a hypoxic and aggressive brain tumor associated with poor patient prognosis and limited treatment options. Such clinical manifestation is, in part, attributable to the highly adaptive mechanisms of the brain cancer cells allowing their metabolic reprogramming within a low oxygen tension tumor microenvironment. In this study, we assessed the transcript levels and the specific roles of the G6P sensing components G6PC3, SLC37A2, and SLC37A4, all found increased in clinical GBM tissues (**Figure 1**) and correlated with decreased overall patient survival (**Figure 2**). Interestingly, G6Pase components G6PC3 and SLC37A4 were also found to be involved in the GBM response to the EMT inducer TGF-β and in the acquisition of a CSC phenotype. With regards to disease progression, our findings further suggest an association between the expression levels of *SLC37A4* and the transition from low-grade glioma state to GBM, adding to their angiogenic and chemoresistance phenotype.

While the importance of glucose metabolism alterations in cancer development and progression is well recognized (43), the specific implication of the G6Pase system components in GBM, particularly with regards to the intracellular conversion of G6P back to glucose, thereby regulating glucose homeostasis and providing a source of energy for cancer cells, has only been inferred within the last decade (22). Dysregulation in the global G6Pase system has been implicated in various cancers, including ovarian cancer and HCC (21, 24), but the specific contributions of the G6PC1-3 and SLC37A1-4 isoforms remains unclear. In addition, G6PC was recently reported as a poor prognosis in cervical cancer and to promote cervical carcinogenesis through EMT progression *in vitro* and *in vivo* (44). These findings shed light over a possible clinical significance of these two G6Pase system components in GBM prognosis.

Consistent with our *in silico* data analysis, functional experiments were performed using several established GBM-derived cell lines in which high expression of *G6PC3*, *SLC37A2*, and *SLC37A4* in comparison to HepG2 hepatoma cells was observed. Specific knockdown of *G6PC3* and *SLC37A4* genes further altered the acquisition of a CSC phenotype, and *G6PC3* as well as *SLC37A4* silencing prevented the TGF-β signal transduction response that led to increased chemotaxis. TGF-β plays an important role in cell metabolism and immunity and can induce a shift in cell metabolism from oxidative phosphorylation to aerobic glycolysis, providing a favorable environment for tumor growth (45). TGF-β also links glycolysis and immunosuppression in GBM as studies have shown that high levels of TGF-β and of its receptors are associated with glioma malignancy and a poor prognosis (46, 47). TGF-β and stem cell markers were found highly expressed around necrotic areas in GBM (48). How *G6PC3* and *SLC37A4* gene silencing alters the crosstalk between G6P sensing and TGF-β signaling remains to be investigated.

Notably, the expression levels of *G6PC3* and *SLC37A4* were found induced in neurospheres, a 3D cell culture model which recapitulates a stem cell-like phenotype associated with increased tumorigenicity and therapeutic resistance (36, 39, 40). This observation suggests that transcriptional manipulation of *G6PC3* and *SLC37A4* levels may contribute to the chemoresistance and invasive molecular signature of GBM and of GBM-derived CSC. The knockdown of *G6PC3* and *SLC37A4* in neurospheres resulted in a significant reduction in the expression levels of CSC markers, including EMT biomarkers, all collectively associated with stemness, self-renewal capacity, and invasive properties of glioma stem cells (49). The impaired stemness properties and reduced invasive features upon knockdown of *G6PC3* and *SLC37A4* again confirm their implication in metabolic reprogramming and suggest their potential as future therapeutic targets to mitigate the aggressive behavior of GBM. Although glycolysis inhibitors are widely used to target such reprogramming, their efficacy in GBM remains unclear, especially within a hypoxic tumor microenvironment (50–52). Inhibition of glycolysis has also been inferred as an effective strategy to eradicate residual brain cancer stem cells that are otherwise resistant to chemotherapeutic agents in their brain-hypoxic niches (53).

Transcriptomic-based studies revealed that upregulated levels of G6PC3 in GBM patients associated with a high-risk group which had significantly poorer survival results (24, 54–57). G6PC3 deficiency in human patients has a broad clinical phenotypic spectrum that involves many organs, including the brain. Its deficiency causes neutropenia in humans and in mice, and is linked to enhanced apoptosis and ER stress (58). The expression of G6PC3 in brain astrocytes, given its low G6P hydrolyzing activity, implicates a novel function for the effective uptake of glucose by astrocytes, and was speculated to allow the ER to function as an intracellular “highway” delivering glucose from perivascular endfeet to the perisynaptic processes (59). Evidence for G6PC3 as a metabolite repair enzyme was also suggested to serve a neuroprotective role in brain to maintain energy-dependent functions, including cognitive processes (60). Previously debated and discounted functions for brain G6PC3 include causing an ATP-consuming futile cycle and a nutritional role involving astrocyte-neuron glucose-lactate trafficking. Interestingly, failure to eliminate a phosphorylated glucose analog led to neutropenia in patients with SLC37A4 and G6PC3 deficiency (61). It was demonstrated that SLC37A4 and G6PC3 collaborated to destroy 1,5-anhydroglucitol-6-phosphate (1,5AG6P), a close structural analog of G6P and an inhibitor of low-*K*_M_ hexokinases, which catalyze the first step in glycolysis in most tissues. Failure to eliminate 1,5AG6P appears to be the mechanism of neutrophil dysfunction and death in G6PC3-deficient mice (62).

In conclusion, our study highlights an original and underestimated potential of G6PC3 and SLC37A4 as prognostic markers and therapeutic targets in brain cancer. The upregulation of these genes in GBM tissues, and additionally in brain CSC models, further suggests their association with stemness properties and invasive characteristics. Whether G6PC3 and SLC37A4 may contribute, in part through G6P sensing processes, to the pro-angiogenic phenotype of GBM remains speculative, but would align with previous studies indicating the involvement of glucose metabolism alterations in angiogenesis regulation (63, 64). Targeting components of the G6Pase system, either involved in the G6P recognition/transport or hydrolysis within the ER, may potentially provide a novel approach to modulate angiogenesis in GBM.

Further studies are warranted to elucidate the precise molecular mechanisms underlying the functional implications of the G6Pase components G6PC3 and SLC37A4 at the protein level in GBM. The targeted delivery and immunoregulatory effects of chlorogenic acid, a potent SLC37A4 inhibitor (28, 65), were recently investigated for the treatment of GBM with promising potential (66, 67). Thus, exploring the therapeutic potential of targeting the G6Pase system components in preclinical models may eventually be valuable in developing novel treatment strategies for GBM. Overall, our findings shed light on a new complex interplay between glucose metabolism, brain cancer progression, and CSC biology.

## Data availability statement

The raw data supporting the conclusions of this article will be made available by the authors, without undue reservation.

## Ethics statement

Ethical approval was not required for the studies on humans in accordance with the local legislation and institutional requirements because only commercially available established cell lines were used.

## Author contributions

ST: Formal Analysis, Investigation, Methodology, Writing – original draft, Writing – review & editing. M-ER: Data curation, Formal Analysis, Investigation, Writing – review & editing. BA: Conceptualization, Formal Analysis, Funding acquisition, Supervision, Writing – original draft, Writing – review & editing.
